# The Role of Neutrophyl-to-Lymphocyte Ratio as a Predictor of Ovarian Torsion in Children: Results of a Multicentric Study

**DOI:** 10.3390/life14070889

**Published:** 2024-07-18

**Authors:** Carlos Delgado-Miguel, Javier Arredondo-Montero, Julio César Moreno-Alfonso, María San Basilio, Raquel Peña Pérez, Noela Carrera, Pablo Aguado, Ennio Fuentes, Ricardo Díez, Francisco Hernández-Oliveros

**Affiliations:** 1 Pediatric Surgery Department, Fundación Jiménez Díaz University Hospital, Avenida de los Reyes Católicos, 2, 28040 Madrid, Spain; 2 Institute for Health Research IdiPAZ, La Paz University Hospital, 28046 Madrid, Spain; 3 Pediatric Surgery Department, Complejo Asistencial Universitario de León, 24071 Castilla y León, Spain; 4 Pediatric Surgery Department, Navarra University Hospital, 31008 Pamplona, Spain; 5 Pediatric Surgery Department, La Paz University Hospital, 28046 Madrid, Spain; 6 Pediatric Surgery Department, Rey Juan Carlos University Hospital, 28933 Móstoles, Spain; 7 Pediatric Surgery Department, Toledo University Hospital, 45005 Toledo, Spain; 8 Pediatric Surgery Department, Villalba University Hospital, 28400 Villalba, Spain

**Keywords:** ovarian torsion, neutrophil-to-lymphocyte ratio, predictive factors, children

## Abstract

Introduction: Pediatric ovarian torsion (OT) is an emergency condition that remains challenging to diagnose because of its overall unspecific clinical presentation. The aim of this study was to determine the diagnostic value of clinical, ultrasound, and inflammatory laboratory markers in pediatric OT. Methods: We performed a retrospective multicentric case–control study in patients with clinical and ultrasound suspicion of OT, in whom surgical examination was performed between 2016–2022 in seven pediatric hospitals. Patients were divided into two groups according to intraoperative findings: OT group (ovarian torsion), defined as torsion of the ovarian axis at least 360°, and non-OT group (no torsion). Demographics, clinical, ultrasound, and laboratory features at admission were analyzed. The diagnostic yield analysis was performed using logistic regression models, and the results were represented by ROC curves. Results: We included a total of 110 patients (75 in OT group; 35 in non-OT group), with no demographic or clinical differences between them. OT-group patients had shorter time from symptom onset (8 vs. 12 h; *p* = 0.023), higher ultrasound median ovarian volume (63 vs. 51 mL; *p* = 0.013), and a significant increase in inflammatory markers (leukocytes, neutrophils, neutrophil-to-lymphocyte ratio, C-reactive protein) when compared to the non-OT group. In the ROC curve analysis, the neutrophil-to-lymphocyte ratio (NLR) presented the highest AUC (0.918), with maximum sensitivity (92.4%) and specificity (90.1%) at the cut-off point NLR = 2.57. Conclusions: NLR can be considered as a useful predictor of pediatric OT in cases with clinical and ultrasound suspicion. Values above 2.57 may help to anticipate urgent surgical treatment in these patients.

## 1. Introduction

Ovarian torsion (OT) is a relatively rare cause of acute abdominal pain in children, with challenging diagnosis due to nonspecific clinical presentation and poor specificity of radiologic tests [1,2]. Severe pain and the sudden onset of the pain are highly suggestive but are not always found. However, delay in establishing the diagnosis and treatment in a timely fashion can result in irreversible ovarian ischemia with functional loss of the ovary [3].

Ultrasound is the mainstay of evaluation for OT in the pediatric population, with a sensitivity of around 80% [4], and some findings related to OT have been described, such as the absence of ovarian Doppler flow, although none of them is pathognomonic [5]. Further, there is near-universal agreement that presence of intra-ovarian blood flow does not exclude torsion [6].

Neutrophil-to-lymphocyte ratio (NLR) has been postulated as an inflammatory biomarker in several ischemic diseases in children such as testicular torsion [7]. In addition, few studies have demonstrated its role as a diagnostic predictor in diseases with significant intra-abdominal inflammatory involvement, such as Henoch–Schönlein purpura and acute appendicitis with peritonitis or intra-abdominal abscess [8,9,10]. Recently, its relationship with adnexal torsion and ovarian tumors in adult women has been investigated, but there is scarce evidence in the pediatric population [11,12]. The aim of this study is to analyze the role of the NLR as a predictor of ovarian torsion in children and adolescents and compare it with other potential clinical, ultrasound, and inflammatory factors.

## 2. Methods

A retrospective multicenter case–control study was performed in patients with clinical and ultrasound OT suspicion, in whom diagnostic laparoscopy was performed at seven pediatric institutions between January 2016 and December 2022. Patients were divided into two groups according to the intraoperative diagnosis of OT, which was defined as the ovary twisting on its axis at least 360 degrees: OT group (ovarian torsion) and non-OT group (no torsion observed).

Demographic variables, clinical features, menstruation status, time since symptoms onset, laboratory variables, ultrasound characteristics, and intraoperative findings were analyzed. Clinical features recorded included the presence of abdominal pain, vomiting, diarrhea or constipation, fever (temperature ≥38.0 °C), dysuria, anorexia, as well as the occurrence of previous similar episodes. Among ultrasonographic features, imaging features (cystic, solid or mixed), ovarian volumen (measured in mL), ovarian volumen ratio (ratio between the volume of both ovaries), absence of Doppler flow, and the presence of pelvic free fluid were reported. Patients with missing data were excluded from the analysis.

Laboratory data were gathered from blood tests conducted in the Emergency Department (ED) upon the patients’ arrival. These tests encompassed a complete blood count, including leukocyte count and absolute counts of neutrophils, lymphocytes, monocytes, basophils, and eosinophils. Additionally, biochemistry parameters such as glucose, fibrinogen, and ion levels were measured, along with C-reactive protein (CRP) levels. NLR was calculated by dividing the absolute number of neutrophils by the absolute number of lymphocytes. Platelet-to-Lymphocyte Ratio (PLR) was obtained by the ratio between the total number of platelets (×10^9^/L) and lymphocytes (×10^9^/L). Systemic Inflammation Response Index (SIRI) was obtained by the following formula: neutrophil × monocytes/lymphocytes [13]. Systemic Immune-Inflammation Index (SII) was obtained by calculating neutrophil × platelets/lymphocytes. Serum levels of human chorionic gonadotropin (hCG), alpha-fetoprotein (AFP), CA 125, CA 19-9, CA 15-3, and carcinoembryonic antigen (CEA) were also measured. The study protocol received approval from the Institutional Review Board (IRB number PI-263-23) and adhered to the principles outlined in the Declaration of Helsinki (2013 revision). Due to the retrospective design of the study and the absence of human samples, informed consent was not required, consistent with institutional ethical standards.

Data were collected using Microsoft Excel software version 2010 (Redmond, WA, USA) and analyzed using SPSS Statistics version 25 (Chicago, IL, USA). Normality of variables was assessed using Kolmogorov–Smirnov and Shapiro–Wilk tests. For normally distributed continuous variables, independent samples t-tests were utilized, with results expressed as mean and standard deviation. Mann–Whitney tests were employed for continuous data not following a normal distribution, and results were presented as median and interquartile range (IQR). Discrete variables were presented as frequency and percentage and analyzed using the Chi-square test or Fisher’s test when applicable. Odds ratios (OR) with 95% confidence intervals were calculated. All statistical analyses were two-tailed, and significance was set at *p* < 0.05. The OT diagnostic yield analysis was performed using logistic regression models, and the results were represented by receiver operating characteristic (ROC) curves by calculating the area under the curve (AUC). The DeLong method was employed to compare these curves [14]. Optimal cut-off values for maximal diagnostic accuracy of each analytical parameter were determined using the Youden index with this formula “sensitivity + specificity − 1” [15].

## 3. Results

A total of 110 patients were included, with a median age at diagnosis of 11.5 years (IQR 8.2–13.6 years), and a median weight of 43 kg (24.5–53.2). In 75 patients, OT was observed during diagnostic laparoscopy (OT group), and in the remaining 35, no OT was observed (non-OT group). Patients in the OT group had a median age of 10.9 years, which was significantly lower than those in the non-OT group (median 12.1 years; *p* < 0.001), although no differences in menstruation status were observed, most of them being in the premenarcheal stage.

Regarding associated symptoms, abdominal pain was the form of presentation in 70% of both groups. Nausea or vomiting was more frequent in the OT group (74% of patients), compared to only 42% in the non-OT group (*p* < 0.001). Hyperthermia, on the other hand, was more frequent in the non-OT group. Time from symptoms onset to ED consultation was also higher in the non-OT group (median 18 h) compared to 12 h in the OT-group (*p* = 0.029). About 20% of patients in both groups had previous episodes. Table 1 shows the demographic and clinical features in both groups.

Concerning ultrasound features (Table 2), the right side was the most affected in both groups. More than half of the lesions had cystic characteristics, followed by mixed lesions. Solid lesions were found in 10–14% of patients, with no differences between the two groups. Patients in the OT group had a higher ovarian volume affected and a higher affected/healthy ovarian volume ratio when compared to those in the non-OT group. In more than 90% of cases, no ovarian Doppler flow was identified. The presence of free fluid in the pelvis was observed in more than half of the patients in both groups, with no differences between them.

In relation to laboratory data, patients in the OT group presented elevated values of leukocytes, neutrophils, NLR, PLR, SIRI, and SII (all *p* < 0.001). There were no differences in monocyte counts, nor in biochemical parameters such as CRP, glucose, fibrinogen, or ionogram. There were also no differences in tumor markers between the two groups. A summary of the laboratory findings is provided in Table 3.

**Figure 1:**
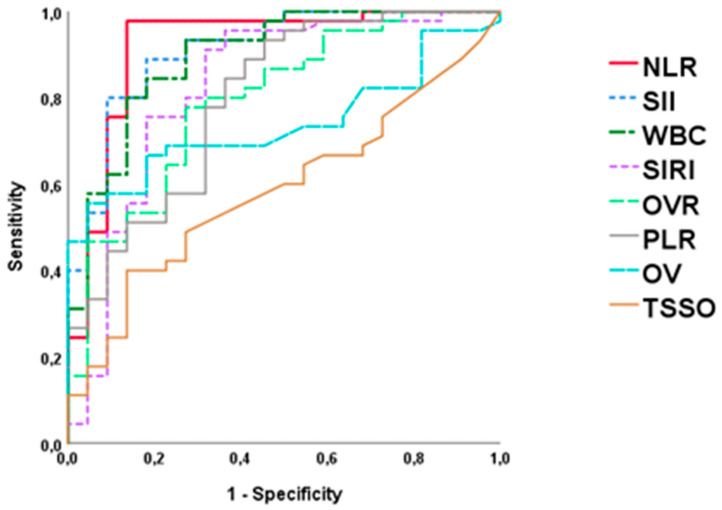
ROC curves for OT intraoperative diagnosis. NLR, Neutrophil-to-Lymphocyte Ratio; SII, Systemic Immune-Inflammation Index; SIRI, Systemic Inflammation Response Index; WBC, White Blood Cells; PLR, Platelet-to-Lymphocyte Ratio; OVR, ovarian volumen ratio; OV, ovarian volumen; TSSO, time since symptoms onset.

Finally, the statistically significant quantitative variables in the univariate analysis were represented by ROC curves (Figure 1, Table 4). NLR was the parameter with the highest AUC (0.918), followed by SII (0.895), WBC (0.874), and SIRI (0.824). The cut-off point of NLR = 2.57 was the one with the highest sensitivity (92.4%) and specificity (90.1%).

## 4. Discussion

This study analyzes different clinical, radiological, and laboratory factors as predictors of ovarian torsion in children. Although clinical symptoms and ultrasound data provide the basis for a diagnosis of suspicion, the NLR has the strongest predictive ability for ovarian torsion in infants. Diagnosis complexity in these patients can cause delays in optimal treatment, and therefore seeking potential predictive biomarkers is important for the prognosis of this disease.

The incidence of ovarian torsion is estimated to be between 0.5 and 2 cases per 10,000 patients, representing approximately 2% to 3% of all visits for abdominal pain in EDs [1,16]. Symptoms can also be very vague and may range from mild to severe pelvic or abdominal pain, nausea, vomiting, or fever, and can even mimic other etiologies of abdominal pain, including acute appendicitis, mesenteric adenitis, constipation, functional ovarian cysts, renal colic, pyelonephritis, and even colitis [17,18]. In postmenarcheal cases, a possible ectopic pregnancy should also be considered [19]. Furthermore, in the pediatric population, reproductive organs lie high in the abdomen and may be difficult to evaluate on physical examination, which may additionally complicate reaching an appropriate diagnosis [20,21]. Abdominal pain is the most common presentation, which was present in more than 70% of patients in both groups. Vomiting and/or nausea are the most common accompanying complaints [4]. In our study, we found that the presence of vomiting was more frequently associated with OT, which may be secondary to the parasympathetic reaction induced by ischemia. In contrast, we observed that hyperthermia was more frequent in patients without OT, more related to abdominal or pelvic infectious causes.

A prolonged interval between the onset of pain and the diagnosis of torsion correlates with a decreased rate of ovarian salvage [16]. However, it is difficult to influence the duration between the first symptoms and consultation in the ED. In our study, patients in the OT group had a shorter time from symptom onset to ED consultation than girls in the non-OT group. The ischemia maintained during the time in the OT may explain that the pain is less bearable in these cases, and that is why the time to go to the ED is shorter. Although ovarian torsion is more common in the postmenarcheal girls due to the increased prevalence of ovarian cysts in these patients, it can also be found in premenarcheal children [22]. Other common acute adnexal pathologies such as simple ovarian cysts with or without rupture are more frequent during menstruation and may be confused with an OT. This may justify the findings found in our study, where the majority of girls in the non-OT group were postmenarcheal, unlike those in the OT group, and consequently, ovarian torsion may still remain a potential diagnosis in both pre- and postmenarcheal girls. The association of vomiting, shorter time from symptom onset and premenarcheal age have been identified by other authors as clinical predictors of OT in girls [23]. However, these studies do not include ultrasound data or inflammatory laboratory parameters.

Ultrasonography is the preferred imaging modality when ovarian pathology is clinically suspected. Signs such as increased ovarian size, the presence of a complex mass, and free fluid can indicate adnexal torsion [5]. However, the ultrasound appearance of ovarian torsion varies depending on factors like the duration and extent of torsion, whether it is complete or incomplete, and the presence or absence of an ovarian mass. Color Doppler sonography has emerged as a potential tool for identifying interruptions in ovarian blood flow in recent years [24,25]. Yet, it is essential to recognize that the presence of vascular flow on Doppler studies does not conclusively exclude torsion, nor does the flow absence confirm OT. Shadinger et al. found arterial flow in 54% and venous flow in 33% of patients with pathologically proven OT [5]. In our study, we found significantly higher ovarian volume and ovarian volume ratio in patients with OT, with no differences in the rates of absence of Doppler flow or pelvic free fluid. We also observed a predominant occurrence of torsion on the right side in both groups, similar to previous studies [26,27,28]. This might be attributed to the presence of the sigmoid colon in the left iliac fossa, which reduces the mobility of the tubal structure and consequently lowers the risk of left adnexal torsion.

Some laboratory data have been tested to predict OT, although several authors found them unhelpful in the diagnostic process [29,30]. CRP levels rise in response to inflammation and tissue necrosis several hours after torsion, making it of little use for early diagnosis [31]. In addition, alternating CRP concentrations during the menstrual cycle have been reported, although they are not usually as high as that encountered in ovarian torsion, but this could potentially make interpretation difficult [31]. In OT, tissue ischemia initiates systemic inflammation that can be quantified in peripheral blood. In adults, markers such as interleukin-6, interleukin-8, tumor necrosis factor-α, and E-selectin have been proposed, but low availability and high cost make their use in clinical practice complicated [31]. In contrast, white blood cell data reflect systemic inflammation, being universally available, quickly analyzed, and cost effective. In this context, the usefulness of NLR as a diagnostic marker of OT has been described, mainly in adult women [11,32]. However, there is scarce experience in pediatric patients. Nissen et al. analyzed laboratory data from 18 girls with OT and 58 controls with ovarian pathology other than torsion and observed that NLR and PLR allowed differentiation between the two cohorts [12]. Nonetheless, they did not include ultrasound data in the analysis, so our present study represents a novelty in this aspect. Furthermore, our study is the first to analyze different inflammatory indices (NLR, PLR, SIRI, SII), which are easily calculated from blood count data, and also compares them with clinical and ultrasound data, similar to what occurs in clinical practice. The results obtained demonstrate that the NLR is the most sensitive and specific predictor for the diagnosis of OT in girls. On the one hand, it is a more objective parameter than clinical data such as time from symptom onset, which is sometimes not very accurate since the presentation sometimes begins with progressive pain. In addition, it avoids the explorer-dependent variability of radiological tests such as ultrasound.

Inflammatory response secondary to ovarian ischemia results in neutrophilia due to chemotaxis and increased release of these cells from the bone marrow to the peripheral blood, which is combined with lymphopenia induced by elevated levels of endogenous cortisol due to ischemia [33]. All this leads to an elevation of the NLR by two combined pathways. This may explain the higher AUC with respect to other inflammatory indices such as PLR, SIRI, or SII that involves monocytes or platelets, as NLR translates the combined cellular response of neutrophilia and lymphopenia. These observations may extend beyond female reproductive organs, as the role of NLR as a predictor of testicular torsion in adolescents has also been recently described [7]. Other authors have described the usefulness of NLR for the differentiation of ruptured ovarian cysts and adnexal torsion, although no analysis of additional inflammatory indices has been performed [34]. Tayyar et al. reported a marked reduction of platelets in patients with OT with comparably unaltered lymphocyte counts, which conditioned a low predictive capacity of the PLR, consistent with the results obtained in our study [35]. In summary, the main advantage of NLR is that it incorporates the informative aspects of two variables representing contrasting immune pathways through the leukocyte subtype ratio, which provides a more accurate depiction of the overall impact of alterations in ovarian torsion. NLR demonstrated superior discriminatory power compared to leukocytes, neutrophils, PLR, SIRI, and SII, as evidenced by higher AUC values in ROC curves analysis.

The main strength of this study is the inclusion of clinical, ultrasound, and laboratory data, which allows ovarian torsion to be analyzed from a holistic perspective, which constitutes a novelty in this aspect. This translates into high applicability, as it includes the same parameters that are used in routine clinical practice in this type of patient. This translates into the need for urgent diagnostic laparoscopy in cases with NLR values are above 2.57, avoiding delays in the management of these patients, while in cases with low inflammatory parameters, initial conservative management can be considered, with subsequent clinical, ultrasound, and analytical reevaluation. However, our study has limitations that should be taken into account. The retrospective design is the main limitation, since it only allows us to analyze the data previously collected in the medical record. In addition, the absence of similar studies in pediatric patients makes it difficult to compare the results obtained. The sample size is also limited, despite the multicenter participation, due to the relatively low incidence of this pathology. Therefore, caution should be adopted when extrapolating or generalizing these results. Prospective studies are required to validate these findings.

## 5. Conclusions

NLR can be considered as a useful predictor of pediatric OT in cases with clinical and ultrasound suspicion. Values above 2.57 may help to anticipate urgent surgical treatment in these patients, avoiding delays in surgical care in these cases. In patients with low inflammatory values, an initial conservative management can be considered, with clinical, ultrasound, and analytical reevaluation. However, prospective studies are required to validate these findings.

## Figures and Tables

**Table 1:** Demographic and clinical features in both groups.

	Group OT (*n* = 75)	Group Non-OT (*n* = 35)	*p*-Value	OR (95%CI)
Age (years); median (IQR)	10.9 (7.3–13.2)	12.1 (11.2–13.7)	<0.001	-
Weight (kg); mean (SD)	35.9 (12.1)	49.4 (16.1)	<0.001	-
Menstruation status; n (%) Premenarchal Postmenarchal	54 (72)	23 (65.7)	0.449	1.34 (0.56–3.17)
	21 (28)	12 (34.3)		
Clinical features; n (%) Abdominal pain Vomiting Diarrhea Constipation Fever Dysuria Anorexia	59 (78.7)	25 (71.4)	0.405	1.47 (0.58–3.69)
	56 (74.7)	15 (42.9)	0.001	3.92 (1.68–9.18)
	2 (2.7)	1 (2.9)	0.577	0.93 (0.08–10.63)
	10 (13.3)	5 (14.3)	0.892	0.92 (0.29–2.94)
	4 (5.3)	6 (17.1)	0.046	0.27 (0.07–1.03)
	5 (6.7)	5 (14.3)	0.195	0.42 (0.12–1.59)
	7 (9.3)	5 (14.3)	0.438	0.62 (0.18–2.10)
Time since symptoms onset (hours); median (IQR)	12 (4–48)	18 (7–72)	0.029	-
Previous episodes; n (%)	15 (20)	6 (17.1)	0.722	1.21 (0.42–3.44)

95% CI, 95% Confidence Interval; OR, odds ratio; IQR, interquartile range; SD standard deviation.

**Table 2:** Preoperative ultrasound characteristics in both groups.

	OT Group (*n* = 75)	Non-OT Group (*n* = 35)	*p*-Value
Affected side; n(%) Right Left	45 (60) 30 (40)	29 (82.9) 6 (17.1)	0.017
Adnexal mass; n(%) Cystic Solid Mixed	38 (50.7) 8 (10.7) 29 (38.7)	23 (65.7) 5 (14.3) 7 (20.0)	0.064
Ovarian volumen (ml); median (IQR)	63 (42–97)	51 (34–78)	0.013
Ovarian volumen ratio; median (IQR)	2.8 (2.1–3.5)	1.9 (1.5–2.4)	0.021
Absence of Doppler flow; n(%)	72 (96)	32 (91.4)	0.325
Pelvic free fluid; n(%)	40 (53.3)	22 (62.9)	0.348

IQR, interquartile range.

**Table 3:** Laboratory variables collected in both groups.

	OT Group (*n* = 75)	Non-OT Group (*n* = 35)	*p*-Value
Leukocytes (×10^9^/L)	10.8 (8.6–13.4)	7.84 (5.73–10.2)	<0.001
Neutrophils (×10^9^/L)	8.58 (6.34–13.22)	3.48 (2.81–6.04)	<0.001
Lymphocytes (×10^9^/L)	1.64 (1.34–1.99)	2.59 (1.84–3.62)	<0.001
Monocytes (×10^9^/L)	0.43 (0.34–0.76)	0.44 (0.32–0.61)	0.942
Platelets (×10^9^/L)	289 (243–329)	295 (240–355)	0.785
NLR	4.6 (3.1–7.9)	1.58 (1.02–2.07)	<0.001
PLR	174.4 (126.8–278.4)	185.7 (99.1–295)	<0.001
SIRI (×10^9^/L)	2.2 (1.1–3.9)	0.6 (0.4–1.2)	<0.001
SII (×10^9^/L)	1385 (796–2542)	391 (265–673)	<0.001
CRP (mg/L)	2.9 (1.5–4.1)	0.9 (0.5–3.5)	0.145
Glucose (mg/dL)	100 (84–114)	98 (83–101)	0.352
Fibrinogen (mg/dL)	337 (255–427)	286 (257–402)	0.589
Ionogram Na+ K+ Cl−	138 (136–140) 4 (3.7–4.4) 105 (103–106)	138 (136–140) 3.9 (3.8–4.2) 106 (105–108)	0.863 0.599 0.242
Tumour markers hCG AFP CA 125 CA 19-9 CA 15-3 CEA	1.2 (0.2–2)	2 (1.2–2)	0.089
	1.7 (1.3–3.2)	1.8 (1.3–10.5)	0.415
	23.7 (15.9–44.7)	14.8 (11.4–26.4)	0.072
	17.7 (9.7–26.0)	16.9 (12.1–35.5)	0.949
	10.4 (9.4–19.8)	10.4 (8.7–15.3)	0.853
	1.0 (0.5–1.2)	0.5 (0.5–4)	0.600

NLR, Neutrophil-to-Lymphocyte Ratio; PLR, Platelet-to-Lymphocyte Ratio; SIRI, Systemic Inflammation Response Index; SII, Systemic Immune-Inflammation Index; hCG, human chorionic gonadotropin; AFP, alpha-fetoprotein; CA 125, cancer antigen 125; CA 19-9, cancer antigen 19-9; CA 15-3, cancer antigen 15-3; CEA, carcinoembryonic antigen.

**Table 4:** Area under the curve (AUC) values, cut-off point, sensitivity, and specificity for OT intraoperative finding.

	AUC	Cut-Off Point	Sensibility	Specificity	*p* Value
NLR	0.918	2.57	92.4	90.1	<0.001
SII (×10^9^/L)	0.895	717.8	88.9	81.8	<0.001
WBC (×10^9^/L)	0.872	9,235	84.4	81.8	<0.001
SIRI (×10^9^/L)	0.824	1.25	75.6	81.8	<0.001
OV (mL)	0.797	58	77.4	75.1	0.025
OVR	0.794	2.3	77.8	72.7	0.012
PLR	0.739	157	73.8	78.2	0.002
TSSO (min)	0.588	296	68.1	79.3	0.245

NLR, Neutrophil-to-Lymphocyte Ratio; SII, Systemic Immune-Inflammation Index; WBC, white blood cells; SIRI, Systemic Inflammation Response Index; OV, ovarian volumen; OVR, ovarian volumen ratio; PLR, Platelet-to-Lymphocyte Ratio; TSSO, time since symptoms onset.

## Data Availability

The data generated and analysed in this study will be made available upon reasonable request to the authors.
